# Intrinsic and Extrinsic Thymic Adrenergic Networks: Sex Steroid-Dependent Plasticity

**DOI:** 10.3389/fendo.2018.00013

**Published:** 2018-01-30

**Authors:** Gordana Momčilo Leposavić, Ivan M. Pilipović

**Affiliations:** ^1^Department of Physiology, Faculty of Pharmacy, University of Belgrade, Belgrade, Serbia; ^2^Immunology Research Centre “Branislav Janković”, Institute of Virology, Vaccines and Sera “Torlak”, Belgrade, Serbia

**Keywords:** thymic noradrenergic innervation, noradrenaline-synthesizing thymic cells, adrenoceptors, sex steroids, thymic involution, thymic programming/reprogramming

## Abstract

The thymus is sexually differentiated organ providing microenvironment for T-cell precursor differentiation/maturation in the major histocompatibility complex-restricted self-tolerant T cells. With increasing age, the thymus undergoes involution leading to the decline in efficacy of thymopoiesis. Noradrenaline from thymic nerve fibers and “(nor)adrenergic” cells is involved in the regulation of thymopoiesis. In rodents, noradrenaline concentration in thymus and adrenoceptor (AR) expression on thymic cells depend on sex and age. These differences are suggested to be implicated in the development of sexual diergism and the age-related decline in thymopoiesis. The programming of both thymic sexual differentiation and its involution occurs during the critical early perinatal period and may be reprogrammed during peripubertal development. The thymic (re)programming is critically dependent on circulating levels of gonadal steroids. Although the underlying molecular mechanisms have not yet been elucidated fully, it is assumed that the gonadal steroid action during the critical perinatal/peripubertal developmental periods leads to long-lasting changes in the efficacy of thymopoiesis partly through (re)programming of “(nor)adrenergic” cell networks and AR expression on thymic cells.

The thymus is organ in which T cells are continually generated in a highly dynamic process comprising T-cell receptor (TCR) gene rearrangement, lineage commitment, and selection (1). These processes are linked to distinct rates of proliferation and cell death by apoptosis (1). With increasing age, the thymus atrophies and declines in functions, the phenomenon termed involution (2). Consequently, thymic generation of naïve T cells declines (2, 3). This leads to the shrinkage of peripheral TCR repertoire and the expansion of memory T cell compartment, i.e., to the changes covered by the canopy term immunosenescence (3–5). At the clinical level, the immunosenescence is associated with a greater susceptibility to infections (6, 7), an impaired response to vaccinations (8, 9), and an increased propensity for malignant diseases (10, 11). In addition, according to the U.S. Center for Disease Control, approximately 80% of aged individuals are afflicted with at least one chronic disease as a result of a declination of immune function. Consequently, factors contributing to the thymic involution and mechanisms of their action are becoming the subject of increased interest in the scientific and healthcare communities alike. It should be emphasized that understanding of the mechanisms underlying thymic involution is important not only for moderating the deleterious effects of immunosenescence, but also for envisaging strategies to “rejuvenate” the immune system. It is noteworthy that even in a significant thymic involution thymopoiesis does not cease completely, so it may be enhanced (12). The thymic “rejuvenation” becomes particularly important after exposure to chemotherapy, ionizing radiation, and some infective agents (e.g., HIV-1) (13).

There is evidence that (i) early perinatal programming of the thymus is crucial for the development of thymic involution, and consequently the efficacy of immune responses from early life through adulthood (14), and (ii) this phenomenon is sexually dimorphic (14, 15). Consistently, sex differences in the organ size, structural organization, and thymopoiesis (14–22), and consequently T-cell immune response (23, 24), have been observed. A more rapid thymic involution was found in male compared with female mice (25). Consequently, adult females have the ability to reject allografts more efficiently, a greater ability to combat various viral and bacterial infections, and superior antitumor responses (23).

There are data indicating that the early programming of the thymus development/involution is shaped by genetic, environmental, and hormonal factors (14). The role of genetic factors has been shown in both mice and rats (26–31). These genetically based differences are suggested to be connected to strain differences in susceptibility to various pathologies involving immune mechanisms (26–30). Environmental factors, such as malnutrition, and exposure to endocrine disruptors, in early postnatal life are also shown to influence the pace of thymic involution (32). Alterations in circulating levels of sex steroids in the critical early postnatal developmental “window” may influence not only sexual dimorphism in structural and functional thymic parameters, but also the timing of thymic involution (15, 21, 33). Furthermore, gonadal steroids may influence sexual dimorphism in thymopoiesis, and the age-related decline in its efficacy through: (i) modulating thymic extrinsic (encompassing noradrenergic nerve fibers) and intrinsic [composed of noradrenaline-synthesizing cells, i.e., “(nor)adrenergic” cells] adrenergic regulatory networks, in terms of their density/noradrenaline content and (ii) adrenoceptor (AR) expression on thymic cells (34, 35). In addition, it should be pointed out that the ablation of gonadal steroids during the peripubertal developmental “window” leads not only to short-term increase in thymic weight and enhancement of thymopoiesis, but also to the long-lasting thymic “rejuvenation” (33).

The central goal of this mini review is to summarize recent findings and current knowledge related to the mechanisms of indirect (nor)adrenaline-mediated action of gonadal steroids on the programming/reprogramming of thymic involution, as its action may be easily controlled by many drugs in use for non-immune indications.

## Thymic Extrinsic and Intrinsic (Nor)adrenergic Regulatory Networks

### Thymic Extrinsic (Nor)adrenergic Network

**Figure 1:**
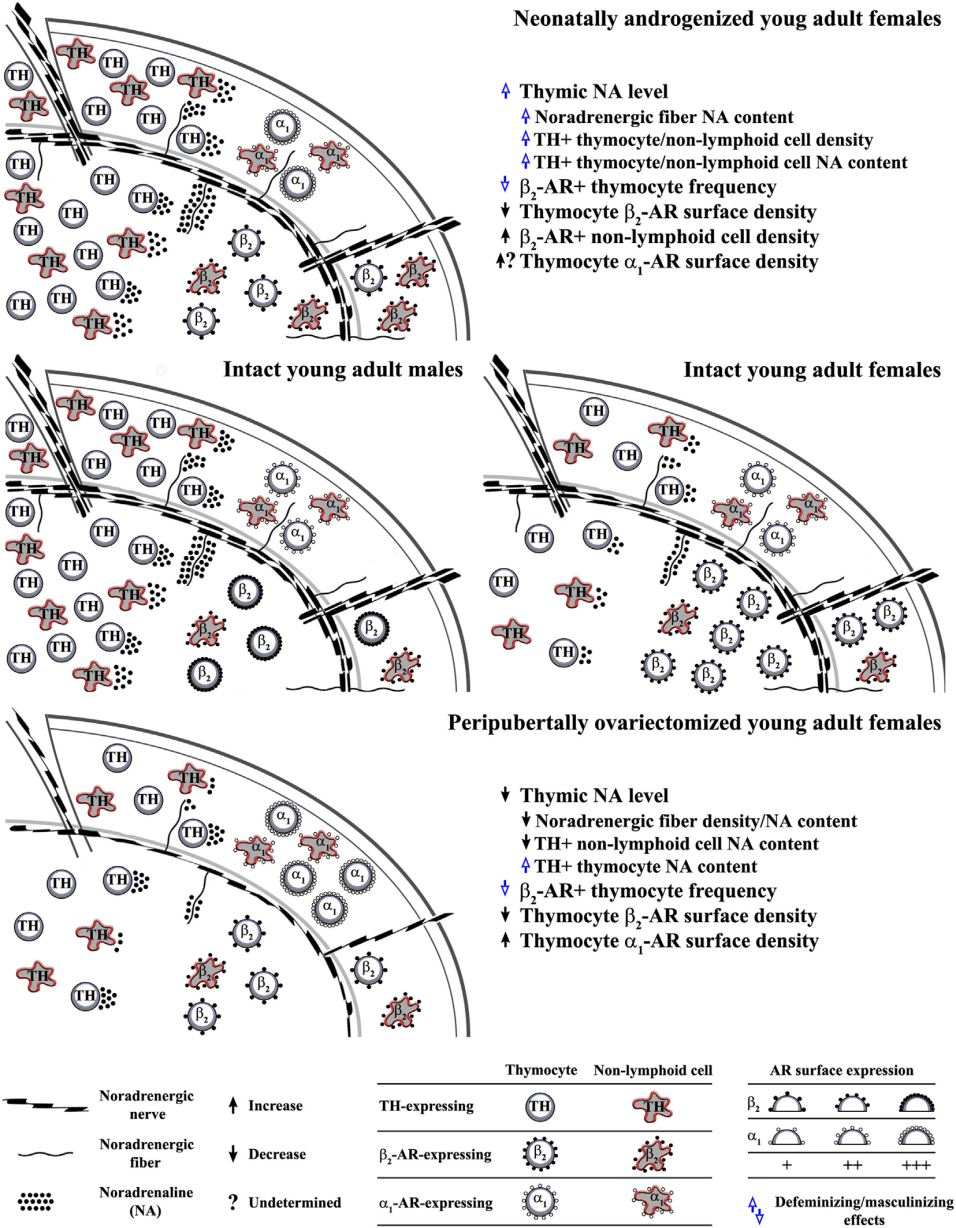
Influence of alterations in circulating ovarian steroid levels in critical developmental periods on programming/reprogramming of thymic extrinsic and intrinsic adrenergic networks. This figure indicates (middle schemes) sex differences in noradrenaline content in noradrenergic nerve fibers and thymocytes, density of tyrosine hydroxylase (TH)-expressing (“adrenergic”) cells, density of β_2_-adrenoceptor (AR)^+^ thymic cells and thymocyte β_2_-AR surface density in young adult rat thymus, and influence of (upper scheme) single injection of testosterone on the third postnatal day to female rats and (lower scheme) ovariectomy in peripubertal period on noradrenergic nerve fiber and thymic “adrenergic” cell density and their noradrenaline content, as well as the density of AR-expressing thymic cells and thymocyte AR surface density in young adult rats.

The thymus receives extensive noradrenergic innervation (36, 37). The varicose noradrenergic fibers terminate in close proximity to thymocytes (37, 38), and various subsets of thymic non-lymphoid (stromal) cells (38–41). In rodents, noradrenergic fibers appear in the thymus in late embryonic period, and their density increases during prepubertal development (42, 43). The data on postpubertal changes in their density are inconsistent (44–50). In advanced age, in rodents of distinct (sub)strains has been observed decrease, increase and lack of changes in thymic noradrenergic nerve fiber density compared with young adult (sub)strain-matched ones (44–50). This inconsistency is most likely linked to (sub)strain and sex-dependent differences in the kinetics of postpubertal changes in thymic noradrenergic innervation. It has also been suggested that the noradrenaline content in thymic nerve fibers, and consequently thymic noradrenaline concentration vary with age (44–50). In addition, both thymic parameters were found to be greater in male than in age-matched female rats (51) (Figure 1).

### Thymic Intrinsic (Nor)adrenergic Network

Many types of mature immune cells synthesize and secrete catecholamines (52–54). The investigations of the expression of tyrosine hydroxylase (TH), the key rate-limiting enzyme in catecholamine synthesis in freshly isolated thymic cells, cultured thymocytes and cells from adult thymic organ culture revealed that thymic cells, including thymocytes, synthetize noradrenaline (34, 51, 55). TH-immunoreactive cells were found across all thymocyte subsets delineated by CD3 expression levels, but their frequency was highest among the most mature CD3^high^ thymocytes (51). In addition, TH-immunoreactive cells were observed in various thymic non-lymphoid cell subpopulations (44, 51). Their density varies across distinct thymic microenvironments. They are frequent at the medullary side of the corticomedullary junction, whereas their density is moderate and poor in the subcapsular cortex, and intracortically/intramedullary, respectively (51). This is important as various thymic non-lymphoid cell subsets are strategically positioned in particular thymic microenvironments to orchestrate thymocyte differentiation/maturation (56). TH immunoreactivity was observed in thymic epithelial cells (TECs) (39, 51, 57–59), macrophages, and dendritic cells (44, 60). In TEC population, TH immunoreactivity was found in neural crest-derived thymic nurse cells (51, 57, 58), type 1 (subcapsular/perivascular), and type 5 (located mainly in corticomedullary region) cells (39, 51, 59). The density of both lymphoid and non-lymphoid TH-immunoreactive cells was shown to be higher in male than in female rats (51) (Figure 1). In addition, the overall noradrenaline content in thymocytes was found to be greater in male compared with female adult rats (51) (Figure 1). Although studies in rat adult thymic organ and thymocyte cultures suggested that noradrenaline from thymic “(nor)adrenergic” cells is implicated in the fine tuning of thymopoisesis (55), a role for thymic intrinsic adrenergic network in thymic homeostasis is still far from being understood. It is noteworthy that intrinsic (nor)adrenergic cellular networks: (i) have also been identified in some other tissues and (ii) suggested to be particularly important under specific conditions, e.g., following sympathectomy, gonadectomy, chronic stress (45, 61–65), as it allows for mainly local regulation of the catecholamine influence (64, 66).

### AR Expression on Thymic Cells

To corroborate modulatory role for noradrenaline in the thymus is the expression of ARs on both thymocytes and thymic non-lymphoid cells. Thymic cells express β_2_- and α_1_-AR (67–70). Their expression is reciprocally regulated during thymocyte maturation (50, 71, 72). The most mature CD3^high^ thymocytes predominantly express β_2_-AR, whereas α_1_-AR expression is predominant on the most immature CD3^−^ thymocytes (50, 60, 70, 72). There is sexual diergism in the expression of β_2_-AR on thymocytes. Immunophenotyping showed the higher frequency of β_2_-AR-expressing cells among thymocytes from female compared with male young adult rats, but lower density of the receptor on their surface (73) (Figure 1). In addition, autoradiographic studies indicated a sexually dimorphic pattern of postnatal changes in the density of β-AR in rat thymus (69). There are no data on sex differences in α_1_-AR expression on thymocytes.

The expression of β_2_-AR was also demonstrated on cortical (aminopeptidase A^+^), and medullary (UEA-1^+^) TECs, CD68^+^ macrophages, and OX62^+^ dendritic cells (44). In addition, α_1_-AR-immunoreactive cells were observed among TECs and macrophages located predominantly in subcapsular/subtrabecular and corticomedullary thymic regions (60). Thymic dendritic cells also express α_1_-AR (74). The subsets of β_2_-AR^+^ and α_1_-AR^+^ non-lymphoid cells were shown to co-express TH (60). Thus, not only paracrine, but also autocrine noradrenaline action may be expected in the thymus.

## Gonadal Steroids and Programming/Reprogramming of the Thymic (Nor)adrenergic Networks and AR Expression

### Early Postnatal Thymic Programming

The thymus is sexually differentiated organ (15). The sexual differentiation in the thymus, as in the brain areas controlling gonadotropin release, occurs during the critical perinatal period, and is governed by sex steroid-dependent mechanisms (15). In addition, the widely accepted organizational/activational hypotesis of the bran development is extended to encompass the thymic differentiation (15). According to the original hypothesis, in the absence of testicular androgens during the critical period (starting at the late prenatal period and continuing, at least, to day 5 postpartum), the areas controlling gonadotropin release develop in a primarily female manner (75–78). Conversely, the presence of testicular androgens leads to their defeminization/masculinization, a phenomenon known as neonatal androgenization (77–79). This postpones sexual maturation and leads to development of non-ovulatory ovaries with estrogen hyporesponsiveness (78, 80, 81). The mechanisms of testosterone action in the brain and thymus are extremely complex, as in both organs it converts into estrogen (15, 75–78), and consequently does not act only through androgen receptors (82). The binding of estradiol to classical estrogen receptor (ER)α or ERβ in the cytoplasm of target cells causes the receptor dimerization and translocation in nucleus, where the dimer associates with various coactivators to enable binding to the estrogen response elements (EREs) in or near the promoters of target genes (83). Estradiol can also influence expression of genes that do not harbor EREs in their promoter regions. In this case, ligand-activated ERs do not bind DNA directly, but through protein–protein interactions with other classes of transcription factors at their respective response elements in promotor region of their target genes (84). In addition, estradiol may act through membrane G protein-coupled ER (GPER, previously termed GPR30) (84). This involves mobilization of diverse signaling pathways and may depend on a number of conditions, like the availability of signal transduction molecules and downstream targets (84).

It was shown that a single injection of testosterone on the third postnatal day enhanced thymic growth and postponed thymic involution in female rats, which normally starts around puberty (85, 86). Accordingly, long-lasting changes in thymopoiesis, mirrored in the enhanced thymocyte differentiation/maturation in adult animals were observed (86). In addition, neonatal androgenization facilitated the generation of CD4^−^CD8^+^TCRαβ^high^ cells, and consequently shifted CD4^+^/CD8^+^ recent thymic emigrant ratio in peripheral blood toward the latter (86). The thymopoietic changes were ascribed to thymocyte overexpression of Thy-1, as its overexpression reduces thymocyte negative selection and favors maturation of CD8^+^ T cells (87). Considering CD8^+^ T cell dominance in the periphery of males when compared with females (23, 88), the previous findings indicate defeminization/masculinization of T-cell compartment in adult neonatally androgenized rats, i.e., speak in favor of a sex steroid role in the sexual differentiation of thymus.

Although aware of the complexity of changes in neuroendocrine-thymic communications in neonatally androgenized rats, in this review we focused on those mediated by catecholamines. Neonatal androgenization was shown to increase thymic noradrenaline concentration in adult rats (35). This mainly reflected the increase in nerve fiber noradrenaline content (35). Consistent with the so-called transsynaptic action of sex steroids on neurotransmitter synthesis (89), the previous finding may be explained by an augmented sympathetic tone in neonatally androgenized rats (90, 91). However, the higher noradrenaline concentration partly reflected the greater density of noradrenaline-synthesizing cells and noradrenaline content per cell (35) (Figure 1). Considering that the circulating level of testosterone was elevated in neonatally androgenized rats (35), this could be associated with data indicating that androgens prominently transactivate TH promoter (92). In light of data from other studies (51), the previous findings suggest thymic defeminization/masculinization in neonatally androgenized rats (Figure 1).

As additional sign of defeminization/masculinization (73), the frequency of β_2_-AR-expressing cells within thymocytes (35) was diminished in neonatally androgenized rats (Figure 1). In addition, neonatal androgenization decreased β_2_-AR density on thymocytes (35) (Figure 1). Given that in many cell types estrogen, acting through classical ERs, upregulates β_2_-AR expression (93, 94), the alterations in β_2_-AR density could reflect estrogen hyporesponsiveness (80, 95). This hyporesponsiveness most likely emerged from the ER interaction with an excess of estrogen (as a result of testosterone aromatization) during the critical period (96, 97). The interaction of receptor with excess ligand in the critical period is shown to cause misprinting substantiated in diminished receptor binding capacity and responsivity in later life (96, 97). The elevation of thymic noradrenaline concentration following the testosterone injection could also impair the efficacy of β_2_-AR signaling (through the hormonal misprinting) (35), leading to the diminished noradrenaline action as the ultimate effect. In favor of this assumption is the increase in Thy-1 expression in adult neonatally androgenized rats (83). Namely, the incubation of murine thymocytes with noradrenaline causes time- and concentration-dependent decreases in the Thy-1 mRNA levels, which are completely preventable by propranolol (98, 99). Moreover, given that: (i) noradrenaline upregulates α_1_-AR expression (100) and (ii) long-lasting α_1_-AR blockade facilitates thymocyte differentiation/maturation toward CD4^+^CD8^−^TCRαβ^high^ cells (70), the contribution of an augmented α_1_-AR signaling (reflecting its increased density and/or noradrenaline concentration) to the thymocyte maturation skewed toward CD4^−^CD8^+^ TCRαβ^high^ cells in adult neonatally androgenized rats cannot be ruled out.

In favor of the role of sex steroids in perinatal programming of thymic noradrenergic networks are also data showing that orchidectomy in the critical perinatal period lowers levels of both neurally- and thymocyte-derived noradrenaline in adult rats and thereby contributes to the deceleration of the thymic involution (34). This is consistent with data indicating that not only in presence of excess ligand in the critical periods, but also in its absence the ligand–receptor connection changes for life (101).

To summarize, the previous findings indicate that alterations in circulating levels of sex steroids in the critical perinatal period may affect the programming of the sexually dimorphic (nor)adrenaline influence on thymopoiesis. However, the molecular mechanisms standing behind this phenomenon remain to be elucidated.

### Peripubertal Thymic Reprogramming

It has been suggested that the hormonal changes occurring at the time of puberty lay the framework for biological differences that persist throughout life (102). In addition, the original organizational/activational hypothesis of sexual differentiation of the brain has been extended to include puberty (76, 103). Namely, ovariectomy in peripubertal period leads to a long-lasting postponement/alleviation of the postpubertal decline in thymopoiesis (33, 104). This could be partly related to ovariectomy-induced changes in thymocyte proliferation (35). Given that the age-related decline in thymopoiesis has been partly related to the rise in the thymic noradrenaline level (44, 50, 105), one may assume that the peripubertal ovariectomy affects thymic adrenergic networks. Indeed, it was shown that it diminishes the thymic noradrenaline level in young adult (2-month-old) rats (45). This reflected the decrease in the density of noradrenergic nerve fibers and noradrenaline content in both noradrenergic nerve fibers and non-lymphoid cells, as thymocyte noradrenaline content increased (45) (Figure 1). These changes were preventable by estrogen supplementation (45). This could be explained by the following facts: (i) estrogen represents the key factor in remodeling of noradrenergic innervation in some other tissues (106) and (ii) is implicated in the regulation of TH expression (107). Estrogen is suggested to regulate TH gene expression through direct genomic effects, as the TH promoter contains several elements, including the activation protein 1 and Sp1/Egr1 motifs that might mediate estrogen action on TH gene (108, 109). The thymic cell type-specific effects of peripubertal ovariectomy on TH expression could be explained by data indicating that estrogen may regulate TH transcription in opposite direction through ERα and ERβ (110). Given that estrogen may influence TH expression trough extragenomic and indirect genomic effects, it may also be supposed that estrogen, through the same ER, may produce opposing effects by interacting with proteins with distinct action on gene transcription in distinct cells (111, 112). In peripubertally ovariectomized rats, the density of noradrenergic nerve fibers and TH-expressing non-lymphoid cells remained lower than in age-matched controls until the age of 11 months (45). On the other hand, thymocyte noradrenaline, which was elevated in 2-month-old peripubertally ovariectomized rats, continued to rise until the age of 11 months (45). In 11-month-old peripubertally ovariectomized rats it was comparable with controls (45). Thus, it seems that the ovariectomy-induced changes are long lasting (45).

In addition, peripubertal ovariectomy in young adult rats diminished the average thymocyte surface density of β_2_-AR, but it increased that of α_1_-AR (reflecting estrogen, and estrogen and progesterone deficiency, respectively) (45) (Figure 1). These changes, despite the rise in circulating estrogen level post-ovariectomy because of extragonadal synthesis (113), remained stable until the age of 11 months (45). This could be related to a decreased sensitivity to estrogen action, as a consequence of peripubertal hormone misprinting. Finally, it is noteworthy that the increased noradrenaline content in thymocytes and diminished frequency of β_2_-AR^+^ thymocytes in young adult ovariectomized rats suggested that peripubertal ovariectomy instigates some signs of thymic defeminization/masculinization (51, 73) (Figure 1).

The putative role of peripubertal orchidectomy in long-lasting reprogramming of the thymic adrenergic networks has not been examined. However, 1 month following peripubertal orchidectomy the changes in both extrinsic and intrinsic noradrenergic networks were similar to those described 1 month following ovariectomy in the same age (44, 45). In addition, an impaired β-AR-mediated influence on thymus led to more efficient thymocyte positive selection/less efficient negative selection, and preferential differentiation/maturation of thymocytes into mature CD4^+^CD8^−^TCRαβ^high^ cells in orchidectomized rats (44), i.e., to a more “feminine” pattern of T-cell development (23).

## Conclusion

In summary, a growing body of evidence indicates that both thymic sexual differentiation and involution are, at least partly, “controlled” during the critical developmental periods by gonadal steroids. In addition, it suggests that the gonadal steroid-mediated thymic (re)programming involves extrinsic and intrinsic noradrenergic regulatory networks and AR expression on thymic cells. The challenge remains to elucidate the molecular mechanisms underlying these gonadal steroid-induced effects. Nonetheless, it may be assumed that (i) alterations in circulating levels of gonadal steroids during the critical developmental periods (either induced endogenously or by endocrine disruptors in the environment) lead to long-lasting effects on thymopoiesis and (ii) pharmacological manipulation with (nor)adrenaline action on thymus may be useful means in preventing/moderating deleterious effects of aging on thymopoiesis.

## Author Contributions

GL wrote the draft version, and IP designed the figure, whereas GL and IP equally contributed to the editing of this manuscript.

## Conflict of Interest Statement

The authors declare that they have not any commercial or financial relationships that could be construed as a potential conflict of interest.
